# Siglec-1 initiates formation of the virus-containing compartment and enhances macrophage-to-T cell transmission of HIV-1

**DOI:** 10.1371/journal.ppat.1006181

**Published:** 2017-01-27

**Authors:** Jason E. Hammonds, Neal Beeman, Lingmei Ding, Sarah Takushi, Ashwanth C. Francis, Jaang-Jiun Wang, Gregory B. Melikyan, Paul Spearman

**Affiliations:** 1 Department of Pediatrics, Cincinnati Children’s Hospital Medical Center, Cincinnati, Ohio, United States of America; 2 Department of Pediatrics, Emory University School of Medicine, Atlanta, Georgia, United States of America; Universitätklinikum Heidelberg, GERMANY

## Abstract

HIV-1 particles assemble and bud from the plasma membrane of infected T lymphocytes. Infected macrophages, in contrast, accumulate particles within an apparent intracellular compartment known as the virus-containing compartment or VCC. Many aspects of the formation and function of the VCC remain unclear. Here we demonstrate that VCC formation does not actually require infection of the macrophage, but can be reproduced through the exogenous addition of non-infectious virus-like particles or infectious virions to macrophage cultures. Particles were captured by Siglec-1, a prominent cell surface lectin that attaches to gangliosides on the lipid envelope of the virus. VCCs formed within infected macrophages were readily targeted by the addition of ganglioside-containing virus-like particles to the extracellular media. Depletion of Siglec-1 from the macrophage or depletion of gangliosides from viral particles prevented particle uptake into the VCC and resulted in substantial reductions of VCC volume. Furthermore, Siglec-1-mediated virion capture and subsequent VCC formation was required for efficient trans-infection of autologous T cells. Our results help to define the nature of this intracellular compartment, arguing that it is a compartment formed by particle uptake from the periphery, and that this compartment can readily transmit virus to target T lymphocytes. Inhibiting or eliminating the VCC may be an important component of strategies to reduce HIV transmission and to eradicate HIV reservoirs.

## Author summary

T lymphocytes and macrophages are the two major cell types involved in HIV replication and transmission events. When a T cell is infected, virus particles assemble and bud from the plasma membrane of the cell. In contrast, infected macrophages develop an intracellular collection of viruses termed the virus-containing compartment or VCC. Many aspects of the formation and function of the VCC remain unclear. Here we show that VCC formation does not actually require infection of the macrophage, but can be reproduced through the addition of virus-like particles or infectious virions to macrophages. HIV-1 particles were captured by the cell surface carbohydrate-binding protein Siglec-1, followed by co-migration of Siglec-1 and captured viral particles to the VCC. Depletion of Siglec-1 from the macrophage prevented VCC formation, and inhibited the ability of infected macrophages to transmit HIV to T cells. Our results help to define the origin of this intracellular compartment, arguing that it is a compartment formed by particle uptake from the periphery. Inhibiting or eliminating the VCC may be an important component of strategies to reduce HIV transmission and to eradicate HIV reservoirs.

## Introduction

Macrophages are readily infected by HIV and make important contributions to AIDS pathogenesis. Currently there is growing interest in this cell type as a potential reservoir for persistent infection and as an important target in efforts to cure individuals of HIV [1–3]. Macrophages are present throughout every organ of the human body, and tissue-resident macrophages may be extremely long-lived, having been derived from progenitor cells during embryogenesis rather than being replaced at short intervals from circulating monocytes [4]. Efforts to understand in detail the interactions between HIV and macrophages are therefore of considerable significance. One of the most enigmatic features of the HIV-infected macrophage has been the presence of the VCC, variously characterized as a source of virus for trans-infection, an immune-protected reservoir, a site of virus assembly, or a site of virus storage following assembly on the plasma membrane. The VCC demonstrates features of the late endosome or MVB compartment, including enrichment of CD9, CD53, CD81, CD82 and MHC class II [5–7]. Unlike late endosomes, however, the compartment is non-acidic and often demonstrates tubular connections that can lead to the plasma membrane [5, 8–11]. The presence of plasma membrane connections to this compartment has led some investigators to refer to the VCC as the intracellular plasma membrane-connected compartment or IPMC [12]. The accessibility of antibodies to this compartment is limited [13, 14] but protection from antibodies may be incomplete [5]. Tetherin plays a role in the formation of the VCC, and tetherin limits HIV transmission from infected macrophages to T cells [15, 16]. We proposed previously that retention of HIV-1 virions by tetherin on the plasma membrane of macrophages contributed to the formation of the VCC, allowing internalization of virions into this compartment [15].

Recently the Gummuluru and Martinez-Picado groups reported an important mechanism utilized by dendritic cells (DCs) to capture, internalize and retain exogenous virus [17–20]. These investigators demonstrated that HIV-1 capture by DCs is dependent on the incorporation of the α-2,3-siaylated gangliosides on the viral membrane. Both GM1 and GM3 contain α-2,3 linkages and were shown to be capable of mediating capture by DCs, while GM3 was more efficient in mediating particle capture at limiting ganglioside concentrations [19, 21]. Virions were captured through an interaction of gangliosides with sialic acid-binding immunoglobulin-like lectin (Siglec-1, also known as CD169), an interferon-inducible member of the I-type lectin receptor family that is present on the plasma membrane of myeloid cells. Depletion of gangliosides from viral membranes or depletion of Siglec-1 in DCs potently inhibited HIV-1 capture and internalization, and also inhibited trans-infection of T cells by mature DCs. The accumulation of particles within intracellular compartments in DCs shares many characteristics with the VCCs of HIV-infected monocyte-derived macrophages (MDMs). In DCs these compartments appear to sequester virus away from the external environment, potentially protecting them from neutralization or other immune defenses. Notably, MDMs have also been shown to express Siglec-1, and capture of virions by MDMs through interaction with sialic acid on gp120 has been proposed as an important mechanism for macrophage infection [22]. Recently it was shown that Siglec-1 on macrophages lining lymphoid sinuses captures murine leukemia virus (MLV)[23] and HIV [24, 25]. In the case of MLV, Siglec-1-mediated capture by macrophages is followed by migration to lymphoid follicles and trans-infection of B cells. Therefore there is increasing evidence that Siglec-1 plays an important role in retroviral particle capture and subsequent transmission events *in vivo*.

Here we examined Siglec-1-mediated virion capture in HIV-infected macrophages, and asked if the Siglec-1-ganglioside interaction plays a role not only in capture of virions but also in the formation of the VCC itself. Exogenous addition of virus-like particles (VLPs) led to their rapid internalization into the VCC in a Siglec-1- and ganglioside-dependent manner, and was not dependent on the presence of the viral envelope glycoprotein. Siglec-1 was highly concentrated, along with tetherin, within VCCs of infected MDMs. Remarkably, VLPs added exogenously became intermingled in the same VCC compartment with viral particles that had originated from the infected macrophage. Furthermore, depletion of Siglec-1 in HIV-1 infected MDMs resulted in a drastic reduction in overall VCC volume and reduced transmission of virus to autologous T lymphocytes. These data demonstrate a prominent role for Siglec-1 in the internalization of HIV-1 to the VCC in infected MDMs, supporting a model in which particle retention on the plasma membrane by tetherin is followed by Siglec-1-driven internalization of particles into the VCC. Furthermore, our results demonstrate that Siglec-1-mediated particle capture and uptake of exogenous HIV-1 particles by uninfected macrophages creates a VCC that is phenotypically identical to that formed in infected macrophages.

## Results

### Interferon stimulation enhances Siglec-1 expression and ganglioside-dependent HIV-1 VLP uptake in human MDMs

**Fig 1:**
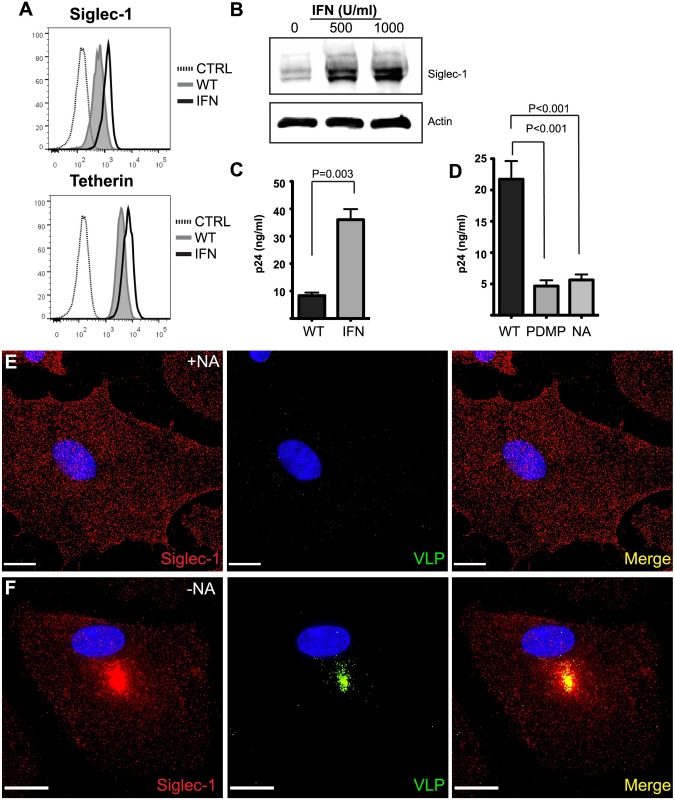
Siglec-1 expression by MDMs is enhanced via IFN exposure and leads to VLP internalization. **(A)** Representative Siglec-1 and tetherin surface expression of GM-CSF matured MDMs together with 24 h IFN alpha stimulation (1000 U/ml). **(B)** Western blot of Siglec-1 and actin upon incubation with increasing amounts of IFN alpha in GM-CSF derived MDMs. **(C)** Enhanced capture of HIV-1 Gag-EGFP VLPs by MDMs stimulated with 1000 U/ml IFN alpha as measured by p24 ELISA. **(D)** MDMs were incubated with 400 ng of sucrose-purified HIV-1 Gag-EGFP VLPs either treated with 1.0 U/μl neuraminidase, untreated or from 293T cultured in the presence of 10 μM PDMP. Cell-associated HIV-1 p24 was quantified by ELISA. **(E)** Sucrose purified, HIV-1 Gag-EGFP VLPs treated with neuraminidase (NA) or untreated **(F)** were added to mature GM-CSF derived MDM cultures on day 8 for 6 hours. Cells were then washed, fixed, immunostained for Siglec-1 (red) and DAPI co-stained. Size bar = 21 μm for **(E)** and 15 μm for **(F)**.

Siglec-1 on the surface of DCs is capable of capturing HIV-1 in a glycosphingolipid-dependent manner [19, 20, 26]. We hypothesized that Siglec-1 may also be responsible for virion capture and subsequent concentration within VCCs of HIV-1 infected monocyte-derived macrophages (MDMs). To address this, we first examined Siglec-1 cell surface levels in MDMs. Siglec-1 was expressed constitutively in human MDMs, and its surface expression was increased upon stimulation with IFN-alpha (Fig 1A, upper plot). The amount of cell surface tetherin was examined in parallel, and was increased approximately 2-fold by IFN stimulation (Fig 1A, lower plot). Total Siglec-1 expression and upregulation by IFN was also apparent by Western blotting, with a 1.9-fold increase in Siglec-1 expression following treatment with 500 U/ml and a 2.7-fold increase with 1000 U/ml of IFN (Fig 1B). Next we asked whether IFN stimulation would enhance the capture and uptake of HIV-1 Gag-EGFP VLPs lacking Env. MDMs were incubated for 1 hour with 100 ng HIV-1 VLP-associated p24/10^5^ cells, vigorously washed, and the amount of p24 internalization measured. Remarkably, IFN stimulation resulted in a 4.3-fold increase in MDM-associated HIV-1 p24 (Fig 1C). This result confirmed that in MDMs, an IFN-stimulated factor or factors was enhancing the capture of exogenously-added HIV VLPs for MDMs, as had been previously shown for Siglec-1 in DCs.

In order to confirm that HIV-1 capture by MDMs requires particle-associated gangliosides as shown for DCs [19, 26], we treated HIV-1 Gag-EGFP VLPs with an α2–3 NeuNAc-specific neuraminidase (NA). We confirmed neuraminidase removal of NeuNAc residues from NA-treated VLPs by staining treated and untreated VLPs with Alexa Fluor 647-conjugated wheat germ agglutinin (WGA) (S1 Fig). Neuraminidase treatment of VLPs resulted in a 3.4-fold reduction in NeuNAc detection as compared with untreated VLPs (S1 Fig). As an additional means of depleting gangliosides on the virion envelope, VLP producer cells were grown in the presence of 10 μM D-threo-1-phenyl-2-decanoylamino-3-morpholino-1-propanol (PDMP), a glucosylceramide synthase competitive inhibitor, 2 days prior and during HIV-1 VLP production. We then evaluated the effect of either neuraminidase treatment or PDMP-mediated depletion of gangliosides on particle uptake by MDMs. Inhibition of glucosylceramide synthesis by PDMP resulted in a 4.6-fold decrease in VLP capture by MDMs, while NA-treatment resulted in a 3.9-fold reduction (Fig 1D). These results establish that GM3 (or potentially GM1) on the VLP surface is critical for the capture and internalization of VLPs by Siglec-1 on macrophages in a manner entirely consistent with published findings for DCs [20, 26]. In order to further understand the differences in particle capture, we examined MDMs 6 hours following exposure to wildtype VLPs or neuraminidase-treated VLPs by fluorescence microscopy. Only a few scattered neuraminidase-treated VLPs were detected on the cell surface of MDMs (Fig 1E). Notably, Siglec-1 displayed a diffuse punctate appearance identical to that of untreated MDMs when exposed to ganglioside-depleted VLPs (Fig 1E). In striking contrast, wildtype VLPs were taken deep into the MDM, and Siglec-1 was found to strongly colocalize with VLPs in this internal compartment (Fig 1F). The position of the VLPs suggested similarities to the VCC of infected macrophages, and the redistribution of Siglec-1 to this compartment suggested to us an active role in VLP internalization.

### Siglec-1 colocalizes with VLPs and is internalized into VCCs of uninfected MDMs

**Fig 2:**
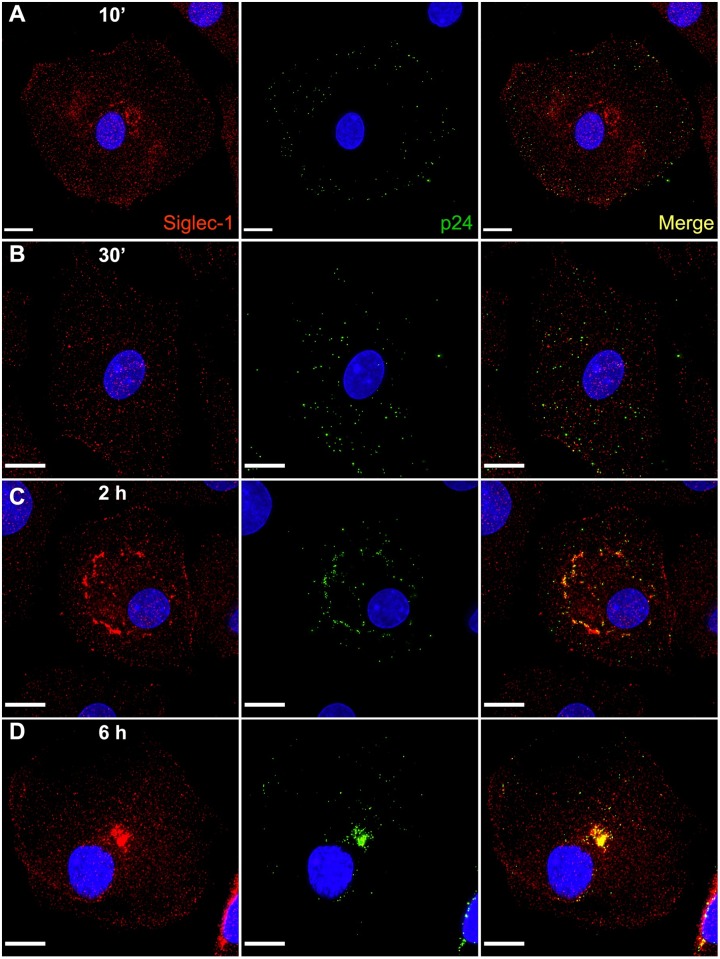
Time course of HIV-1 Gag-EGFP internalization within MDMs and colocalization with Siglec-1. **(A-D)** 400 ng of sucrose-purified HIV-1 Gag-EGFP VLPs were added to MDM cultures and allowed to attach and be internalized from 10’ to 6 hours. At the indicated times, MDMs were washed, fixed in 4% PFA and immunostained with Siglec-1 (red, mAb clone 7–239, AbD Serotec) and DAPI co-stained. Shown are cells representative of the populations examined at each timepoint. Size bars = 10 μm.

We next performed time course experiments to examine the capture and internalization of VLPs by Siglec-1. HIV-1 VLPs captured by MDMs were internalized and concentrated centrally through a series of sequential steps, including initial attachment (10 minutes, Fig 2A), internalization into small colocalizing puncta (30 minutes, Fig 2B), organization into a ring-like structure surrounding what is assumed to be the ER/TGN (2 hours, Fig 2C), and finally concentration into a central perinuclear location (6 hours, Fig 2D). Siglec-1 colocalization with captured HIV-1 Gag-EGFP VLPs was readily apparent throughout each stage of capture, internalization, and concentration into a perinuclear compartment (Fig 2A–2D). In order to examine the capture and internalization of Gag-EGFP VLPs by individual MDMs over time, we performed live cell confocal microscopy. The rapid centripetal movement of exogenous VLPs into the VCC of MDMs is dynamically illustrated in S1 and S2 movies. Together these data indicate that Siglec-1 and VLPs move together from the plasma membrane to the VCC, and show that the internalization of VLPs occurs over a period of minutes to a few hours, resulting in the formation of a concentrated central compartment where both VLPs and Siglec-1 are concentrated.

### Capture and internalization of HIV-1 VLPs is attenuated by Siglec-1 knockdown in MDMs

**Fig 3:**
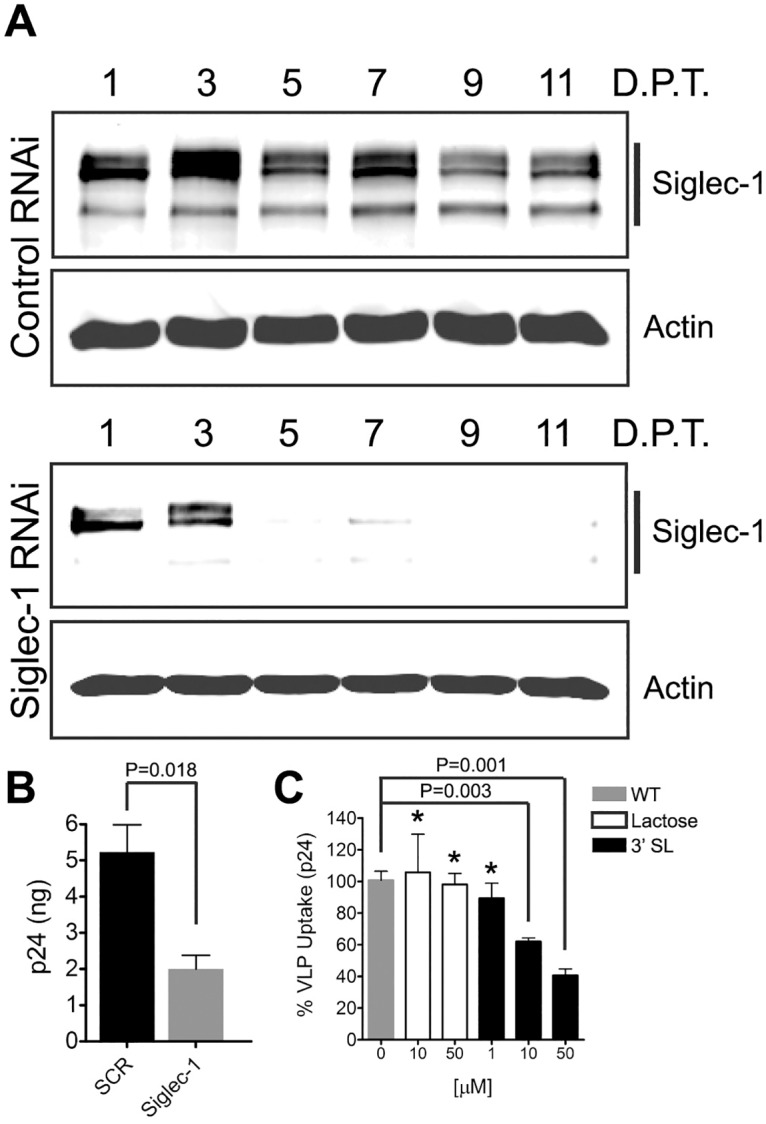
Siglec-1 RNAi interference and competitive inhibition by a GM3 glycan mimetic inhibits HIV-1 VLP internalization by MDMs. **(A)** MDMs were transfected with 60 nM control or Siglec-1 siRNA on day 8 after plating. Cell lysates were harvested and analyzed by Western blotting for Siglec-1 and actin expression. **(B)** MDMs were transfected with either control or Siglec-1 siRNA and 5 days later incubated with 400 ng of HIV-1 Gag-EGFP VLPs for 2 hr. Cells were washed and cell lysate p24 concentration determined by ELISA. **(C)** MDMs were pre-treated at RT with lactose or 3’-sialyllactose for 1 hour. Subsequently, MDMs were incubated with 400 ng of HIV-1 Gag-EGFP VLPs in the presence of compound for an additional hour. MDMs were then washed, cell lysates harvested and cell-associated p24 measured by ELISA. Error bars represent standard deviation; asterisks depict significant differences as measured by unpaired *t*-test.

In order to further demonstrate the functional significance of Siglec-1 in HIV-1 capture and internalization, MDMs were transfected with Siglec-1-specific or control siRNAs. Siglec-1 expression was analyzed by Western blotting of harvested cell lysates over an 11 day time-course following siRNA transfection. Siglec-1 levels were reduced by more than 75% by day 5 post-transfection and by 91% at day 11 (Fig 3A). Siglec-1 expression in MDMs was largely unaffected by transfection with control siRNA (Fig 3A, control panel). In some experiments, MDMs were also treated with tetherin siRNA as described previously [15](S2 Fig). We then performed VLP capture experiments in control and Siglec-1 siRNA-treated MDMs. Knockdown of Siglec-1 reduced VLP capture efficiency to 38% of control, a 2.6-fold reduction (Fig 3B). To further demonstrate sialyllactose-dependent Siglec-1 capture of HIV-1 VLPs in MDMs, competitive inhibition experiments were performed. MDMs were treated with either lactose as a control or with the GM3 polar head group mimetic 3’-sialyllactose at concentrations ranging from 1 to 50 μM for 30 minutes prior to the addition of HIV-1 VLPs. VLPs were then incubated in MDM culture for an additional 2 hours at 37°C. Addition of lactose to the culture medium had no effect on MDM VLP capture, whereas 3’sialyllactose inhibited VLP capture by 60%, a 2.4-fold reduction (Fig 3C). Next, we performed imaging of MDMs exposed to HIV Gag-EGFP VLPs to determine the effect of Siglec-1 knockdown on particle uptake. Control (scrambled) siRNA treatment did not inhibit VLP uptake and colocalization with Siglec-1 (S3A Fig), while only few scattered VLPs were visible following depletion of Siglec-1 (S3B Fig). The volume of the VCC, measured as EGFP voxels, was dramatically reduced following Siglec-1 depletion (S3C–S3E Fig, Siglec-1 siRNA). In control siRNA-treated MDMs, the volume of the VCC increased over time (measured at 30 minutes, 2 hours, and 6 hours, S3C–S3E Fig). Together, these data indicate that like DCs, MDMs capture and internalize HIV-1 VLPs predominantly in a Siglec-1 dependent manner.

### Siglec-1 is concentrated in VCCs of infected macrophages

**Fig 4:**
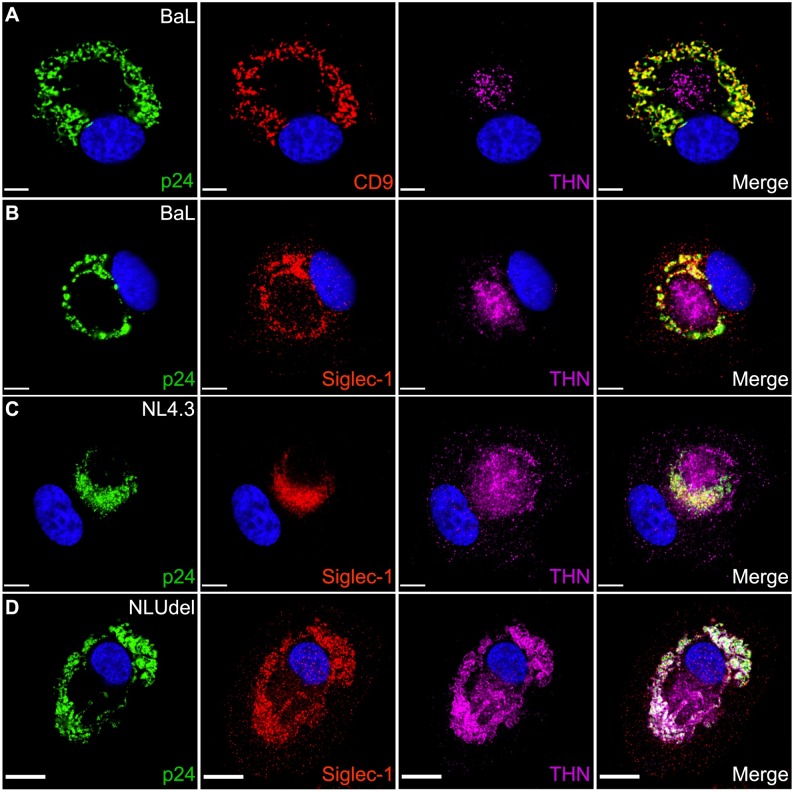
Siglec-1 colocalizes with p24 in VCCs of HIV-1-infected MDMs. **(A)** MDMs were infected with a biological stock of HIV-1 BaL at a TCID_50_ of 0.5/cell. 10 days post-infection cells were fixed and immunolabeled for HIV-1 p24 (green), CD9 (red), tetherin (magenta) and DAPI co-stained. **(B)** Same immunostaining profile as (A) with the exception of Siglec-1 (red) in place of CD9. Size bar = 10 μm. **(C)** MDMs were infected with VSV-G pseudotyped NL4.3 and NLUdel **(D)** at a TCID50 of 2.0/cell. 10 days post-infection cells were fixed and immunostained as described in **(B)**. All size bars = 10 μm. Representative maximum intensity deconvolved images shown.

Because VLP capture created a compartment in uninfected MDMs that resembled the VCC, we next asked if Siglec-1 is concentrated within the VCC of HIV-1-infected MDMs (in the absence of any non-infectious VLP addition). MDMs were infected at an MOI of 0.5 with either the macrophage-tropic BaL strain of HIV, or with VSV-G-pseudotyped NL4.3 or NLUdel, and cultures maintained for 10 days prior to imaging. As expected, CD9 co-localized extensively with p24 in large, multi-vesicular compartments when MDMs were infected with the macrophage-tropic BaL strain of HIV. Downregulation of tetherin by BaL Vpu was apparent, as tetherin signal was localized to a compartment consistent in terms of location with the *trans*-Golgi network with relatively low-level presence in the VCC (Fig 4A). Remarkably, Siglec-1 was found to be highly concentrated within the VCC (Fig 4B). Colocalization of Siglec-1 and p24 was consistently observed in BaL-infected MDMs from multiple donors. We expanded this analysis to include VSV-G-pseudotyped HIV-1 molecular clones NL4.3 and its *vpu*-deficient partner, NLUdel, in order to allow comparison with prior work and to define colocalization with tetherin. Siglec-1 colocalized significantly with HIV virions in NL4.3-infected MDMs (Fig 4C). Tetherin was more prominent within the VCC in NL4.3-infected MDMs as compared with BaL (Fig 4B and 4C). Within NLUdel-infected MDMs, a striking colocalization between Siglec-1, p24 and tetherin was observed (Fig 4D). Measures of colocalization applied to multiple images confirmed the results represented in Fig 4. For BaL-infected MDMs, p24 colocalization with Siglec-1 was high at 86.0 ± 6.9%, while tetherin colocalization was only 16.5 ± 13.7% (see Experimental Procedures for colocalization methods). For NL4.3 and NLUdel-infected MDMs, p24 colocalization with Siglec-1 was also high at 93.9 ± 8.2 and 86.1 ± 12.7%, respectively. In contrast to results observed with BaL infected MDMs, p24/tetherin colocalization was markedly higher for NL4.3 and NLUdel infected MDMs at 64.6 ± 20.9 and 78.7 ± 20.9%, respectively. We attribute the differences observed in p24/tetherin colocalization observed from BaL and NL4.3 infected MDMs to allelic differences in the efficiency of counteraction of tetherin by BaL vs. NL4.3 *vpu* genes, as NL4.3 *vpu* has been shown to be relatively weak compared to many naturally-occurring *vpu* genes [27]. These data overall demonstrate that Siglec-1 is highly concentrated in the VCC of infected macrophages, consistent with what had been observed upon addition of non-infectious VLPs, implying a role for Siglec-1 in virion capture and VCC formation during infection of macrophages.

### Siglec-1 is necessary for VCC formation in HIV-1-infected MDMs

**Fig 5:**
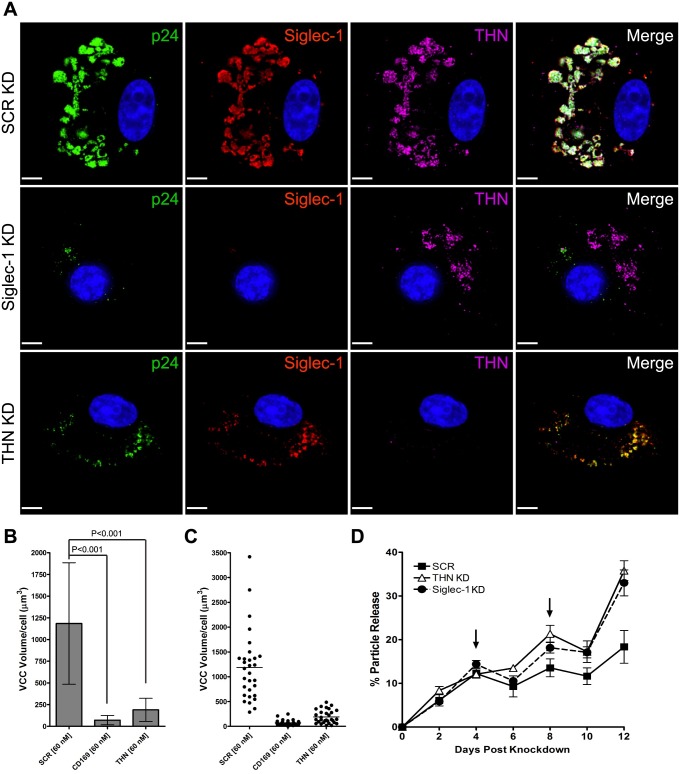
Siglec-1 RNAi reduces VCC volumes in HIV-1 NLUdel-infected MDMs. **(A)** MDMs were infected with VSV-G pseudotyped NLUdel. On the following day, MDMs were transfected with 60 nM scrambled, Siglec-1 or tetherin siRNA. At day 10 post-infection, MDMs were washed, fixed with 4% PFA and immunostained for p24 (green), Siglec-1 (red), tetherin (magenta) and DAPI co-stained. Size bars = 10 μm. **(B)** P24 VCC volume/cell from 30 NLUdel infected MDMs from each group were quantified using the Volocity 6.3 measurement module. Mean +/- SD shown for each RNAi group. **(C)** Scatter plot displaying individual p24 VCC volume measurements and mean from **(B)**. **(D)** p24 release from Siglec-1, tetherin or control siRNA treated and NLUdel-infected MDMs was assessed over 12 days using a p24 ELISA. The efficiency of particle release is plotted as percentage of extracellular p24/total p24 from 3 experiments, with standard deviations indicated. Arrows indicate two timepoints when the media was changed, resulting in a transient drop in the ratio of extracellular p24/total p24.

Based on the strong colocalization data demonstrating concentration in the VCC of infected MDMs, we examined the effect of Siglec-1 depletion on the formation of the VCC. We first employed NLUdel, as the effect of tetherin on VCC size is enhanced in the absence of Vpu. MDMs were infected overnight with VSV-G-pseudotyped NLUdel at a TCID_50_ of 2.0/cell. On the following day, MDMs were treated with 60 nM of either control, Siglec-1 or tetherin-specific siRNAs, and samples fixed on day 10 post-infection. VCC volume was quantified by measuring the volume of intracellular HIV-1 p24 immunostained areas. Control siRNA-treated MDMs were indistinguishable from untreated MDMs, containing large, intracellular accumulations of p24 that colocalized strongly with both Siglec-1 and tetherin (Fig 5A, top row of images). Siglec-1 siRNA-treatment resulted in a substantial reduction in VCC volume within HIV-1 infected MDMs, measured as 6.1 ± 4.3% of control (Fig 5A, middle row and quantified in 5B and 5C). Previous work in our lab by Chu and coworkers identified tetherin’s role in enhancing VCC formation in HIV-1 infected MDMs [15]. Therefore, we also quantified VCC volumes from tetherin siRNA-treated MDMs. Tetherin siRNA-treated MDMs infected with NLUdel also exhibited a large decrease in VCC volumes, although somewhat less than that observed with Siglec-1 siRNA-treated MDMs (6.3 v 16.4-fold reduction, respectively) (Fig 5A, Tetherin knockdown row and 5B and 5C). Furthermore, the remaining p24 signal in tetherin siRNA-treated, HIV-1-infected MDMs colocalized significantly with Siglec-1 (Fig 5A, tetherin knockdown). The average VCC volume quantified from 30 control siRNA-treated HIV-1 infected MDMs on day 10 post-infection was 1185 μm^3^, whereas the average VCC volume of Siglec-1-depleted MDMs was radically reduced to 72.3 μm^3^ (Fig 5B and 5C). Tetherin knockdown in NLUdel-infected MDMs also resulted in a significant VCC reduction, averaging 190.1 μm^3^. The VCC volume distribution of control siRNA-treated MDMs was large, ranging from 286.4 to 3419 μm^3^. VCC volume ranges for Siglec-1 and tetherin siRNA-treated MDMs were greatly reduced, ranging from 248.1 to 2.2 and 486.1 to 19.9 μm^3^, respectively (Fig 5C). These data demonstrate that reduction in Siglec-1 is dramatically associated with a reduction in the formation of the VCC in HIV-infected MDMs, similar to but to an even higher level than depletion of tetherin.

It is logical to expect that if less virus is internalized by Siglec-1 and tetherin, there would be greater amounts of virus released into the cellular supernatant. Indeed this was the case. We measured p24 within NLUdel-infected MDMs and in the supernatant over time. Depletion of either Siglec-1 or tetherin resulted in a significantly higher percentage of released/accumulating virions in the cell supernatant over time (Fig 5D). Results here are shown as % release, calculated as total p24 in supernatants/p24 in supernatants + cells.

We repeated these siRNA knockdown experiments using the primary HIV-1 isolate BaL. BaL-infected MDMs treated with control siRNA contained large, concentrated areas of HIV-1 p24 immunostaining on day 10 post-infection consistent with VCC morphology. Siglec-1 colocalization with HIV-1 p24 signal was nearly complete (S4 Fig, top row of images). Within BaL-infected MDMs, tetherin signal was largely found in locations consistent with the TGN rather than the VCC, consistent with the presence of active Vpu expression and tetherin downregulation from the plasma membrane and from virion assembly sites. Depletion of Siglec-1 in HIV-1 infected MDMs via siRNA-treatment resulted in a substantial reduction in VCC volume (S4 Fig, middle row). Alterations to VCC morphology were noted to include loss of concentration and smaller, individual p24-positive compartments. Tetherin siRNA depletion also resulted in smaller whole cell VCC volumes on average, though the change was less dramatic than that seen with NLUdel (S4 Fig, lower row). Interestingly, and not unexpectedly, remaining p24 signal within VCCs of tetherin siRNA-treated, BaL-infected MDMs significantly colocalized with Siglec-1. Taken together, these data demonstrate a critical role for Siglec-1 in the formation of the VCC of infected MDMs.

### Exogenously-added HIV-1 VLPs are transported into the VCCs of HIV-1- infected MDMs

**Fig 6:**
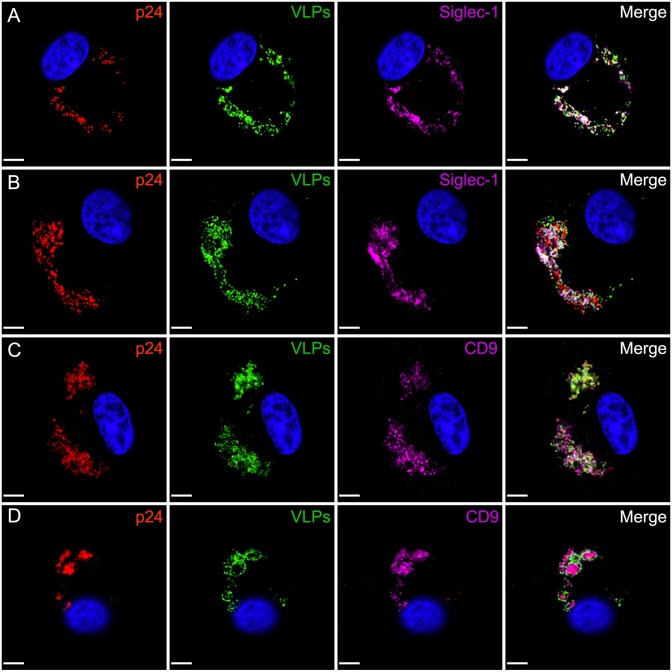
Exogenous HIV-1 VLPs colocalize with Siglec-1 and p24 in the VCC of HIV-1 BaL-infected MDMs. **(A-B)** MDMs were infected with primary HIV-1 isolate BaL at a TCID50 of 0.5/cell. On day 9 post-infection 400 ng of exogenous HIV-1 Gag-EGFP VLPs were added to the culture overnight. On day 10, MDMs were washed, fixed in 4% paraformaldehyde and immunostained for p24 (red), Siglec-1 (magenta) and DAPI co-stained. **(C-D)** MDMs were infected and exogenous HIV-1 VLPs added as described in (A-B). MDMs were washed, fixed in 4% PFA and immunostained for p24 (red), CD9 (magenta) and DAPI co-stained. Size bars = 10 μm.

The VCC has been defined as a compartment arising only in HIV-infected macrophages, rather than as a compartment that could be formed upon addition of viruses exogenously. We next asked if exogenously added HIV-1 Gag-EGFP VLPs captured by BaL-infected MDMs were destined to be transported into the same compartments occupied by particles arising within the infected macrophage (the VCC). In order to perform this experiment, we designed a method to distinguish exogenous VLPs from endogenous, infectious virions by fluorescence microscopy. The murine anti-p24 mAb, KC57-RD1 (Beckman Coulter), fails to recognize immature HIV-1 Gag-GFP VLPs under our immunostaining protocols, while mature HIV-1 virions are efficiently detected by this reagent (S5 Fig). Remarkably, HIV-1 Gag VLPs were substantially concentrated together with endogenous virions in VCCs (Fig 6A and 6B). Both exogenous VLPs and endogenous BaL virions colocalized significantly with Siglec-1 in VCCs. The extent of colocalization between exogenous VLPs and BaL p24 was on average 73 ± 12.9%. Remarkably, 75 ± 15.7% of exogenous VLPs and 70 ± 10.6% of endogenous BaL p24 colocalized with Siglec-1 within BaL-infected MDMs. To further prove that this compartment is identical to the VCC previously described, the compartment was shown to concentrate CD9 together with both infectious virions and VLPs (Fig 6C and 6D). Interestingly, while near-complete colocalization was demonstrated in the VCC of the majority of cells examined, the internalized VLPs sometimes seemed to surround existing VCC material (as in Fig 6D), suggesting that the VLPs were being added to the outer layer of the pre-existing compartment prior to any further mixing. These data indicate that MDMs capture exogenous HIV-1 and internalize these particles into the VCC. In other words, the compartment where virions from the infected macrophage reside, the VCC, is identical to the compartment to which exogenously-added VLPs are delivered to through the Siglec-1-ganglioside interaction.

### Electron microscopic evidence of delivery of exogenous VLPs into the VCC of infected macrophages

**Fig 7:**
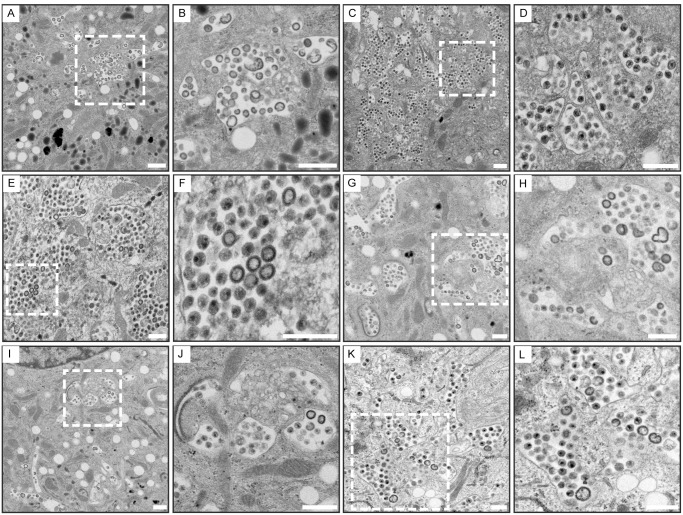
Electron microscopic evidence of colocalization of exogenous HIV-1 VLPs and endogenous mature virions within the VCC in HIV-1-infected MDMs. **(A)** 400 ng of HIV-1 Gag-EGFP VLPs (ratio of WT:Gag-EGFP 1:1) were added to mature MDMs and allowed to internalize overnight prior to harvest and subsequent processing for transmission EM. **(B)** Enlargement of white dotted boxed area from **(A)**. **(C)** NLUdel infected MDMs at day 10 post-infection displaying typical VCC morphology incorporating large amounts of mature HIV-1. **(D)** Enlargement of white dotted boxed area from **(C)**. **(E)** MDMs were infected with NLUdel at a TCID50 of 2.0/cell. On day 9 post-infection, 400 ng of HIV-1 Gag-EGFP VLPs (ratio of WT:Gag-EGFP 3:1) were allowed to internalize overnight prior to harvest and processing. **(F)** Enlargement of white dotted boxed area from **(E)**. **(G-L)** Same procedure as **(E)**, except HIV-1 Gag-EGFP VLPs produced at a WT:Gag-EGFP ratio of 1:1 in order to simplify identification of exogenous VLPs. In each paired image, the enlarged section is indicated by a white broken line in the preceding image. Images were acquired on a 120 kV Hitachi H-7500 transmission electron microscope. Size bars = 500 nm.

In order to further confirm the delivery of VLPs into the VCC, we added Gag VLPs to infected MDMs as before, followed by fixation and preparation for transmission electron microscopy. HIV-1 Gag-EGFP VLPs added exogenously to mature uninfected MDMs were efficiently internalized into compartments morphologically resembling VCCs (Fig 7A and 7B). These particles can be clearly distinguished from mature virions by their immature and sometimes irregular Gag core. In contrast, the majority of particles in the VCC of control infected MDMs demonstrated dense conical cores indicative of mature virions as expected (Fig 7C and 7D). To assess whether exogenous HIV-1 VLPs are delivered to the VCC alongside endogenous HIV-1 in infected MDMs, HIV-1 VLPs were added to both NLUdel (Fig 7E and 7F) and BaL-infected (Fig 7G–7L) MDMs. In order to further accentuate the difference between the VLPs and the native mature particles, VLPs employed in the experiments shown in Fig 7G–7L were produced at a ratio of wild-type to Gag-GFP of 1:1 (rather than 3:1), producing VLPs with a very irregular core morphology. In both scenarios, exogenous VLPs were delivered into compartments containing mature HIV-1 virions (Fig 7E–7L). The compartments bearing mixed virions and VLPs were often deep in the cell as shown in Fig 7I, and displayed complex shapes as well as areas with a tubular appearance (Fig 7I and 7J). These experiments confirmed to us that the addition of VLPs to infected MDM cultures led to internalization of the VLPs into the pre-existing VCCs of infected MDMs.

### Uptake of infectious HIV-1 particles into the VCC of uninfected macrophages

**Fig 8:**
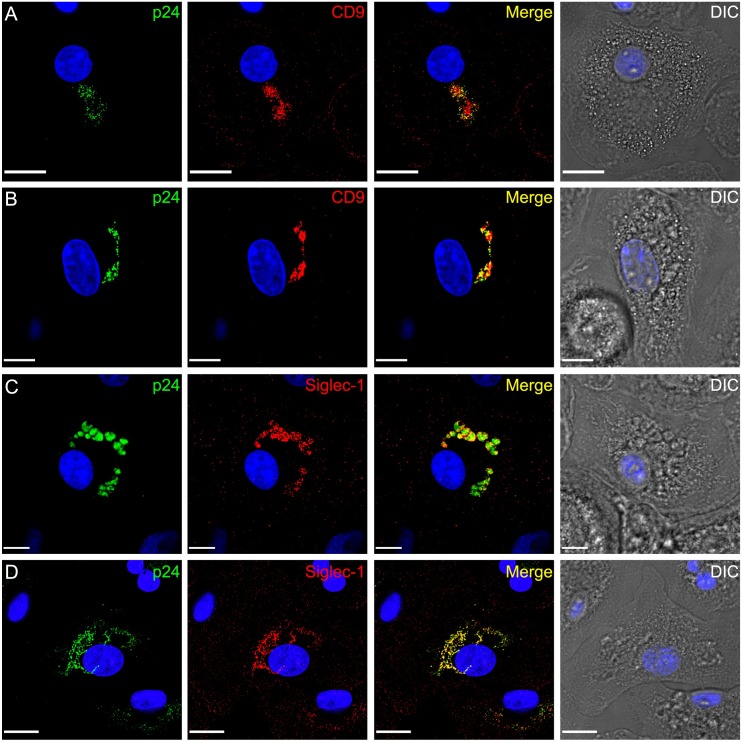
Uptake of infectious virus into VCCs of human macrophages. NL delta Env virus was pseudotyped with macrophage-tropic BaL Env and added to MDM supernatants at 120 TCID50 per MDM in the presence **(B, D)** or absence **(A, C)** of 10μM concentration of the entry inhibitor BMS-626529. Cells were fixed and immunostained for p24 and CD9 **(A, B)** or p24 and Siglec-1 **(C, D)** 24 hours following addition of virus.

Our results above showed that HIV-1-infected macrophages take up and deliver non-infectious VLPs into a VCC containing infectious virions. We next asked if infectious HIV-1 particles will be taken up in a similar manner. We hypothesized that the low amount of CD4 on the macrophage cell surface may not allow receptor binding and fusion of all virions, and that Siglec-1-mediated capture may allow uptake of infectious virions from the surrounding media just as had been seen with VLPs. Macrophages were exposed to higher levels of viral particles than used in typical infection experiments in order to visualize uptake and VCC formation exactly as we had done for VLP uptake (i.e. 100 ng p24 of NLΔEnv virus pseudotyped with BaL Env /1 x 10^5^ cells). As a control, we added BMS-626529, a small molecule that binds gp120 and prevents conformational changes in Env that are required for attachment and entry [28, 29], to some wells. 10μM BMS-626529 was able to completely block infection of TZM-bl cells by NLΔEnv/BaL virus (S6A Fig). Remarkably, infectious virions were taken into an intracellular compartment with characteristics of the VCC as shown by CD9 staining (Fig 8A). Uptake into this compartment occurred in both the absence (Fig 8A) or presence (Fig 8B) of blockade of gp120-CD4 interactions. Staining for Siglec-1 revealed striking colocalization with p24 in this compartment (Fig 8C and 8D). NLΔEnv/BaL p24 colocalized with Siglec-1 with a colocalization coefficient (M1, green/red pixels) of 64% ± 3.1% in the absence of inhibitor vs. 69% ± 3.4% in the presence of inhibitor (S6B Fig). The extent of Siglec-1/p24 (M2, red/green pixel) colocalization was slightly lower overall but similar between treatment groups (S6C Fig). We conclude that the presence of a fusion-competent envelope on exogenous virions does not prevent uptake of virions into the VCC, likely due to inefficient fusion in this cell type that exhibits low levels of surface CD4. The volume of the VCCs formed in presence of BMS-626529 was 38.9 ± 3.1 μm^3^, as compared with 65.2 ± 7.3 μm^3^ in absence of inhibitor (S6D Fig). Electron microscopic analysis of macrophages fixed 24 hours following addition of NLΔEnv/BaL virus revealed intact, mature virions within convoluted intracellular membranous compartments consistent with a classical VCC (S7A and S7B Fig). We could not discern a morphologic difference in this compartment conferred by the presence of the attachment/fusion inhibitor (S7C and S7D Fig).

### Siglec-mediated uptake of virions into the VCC enhances trans-infection of T lymphocytes

**Fig 9:**
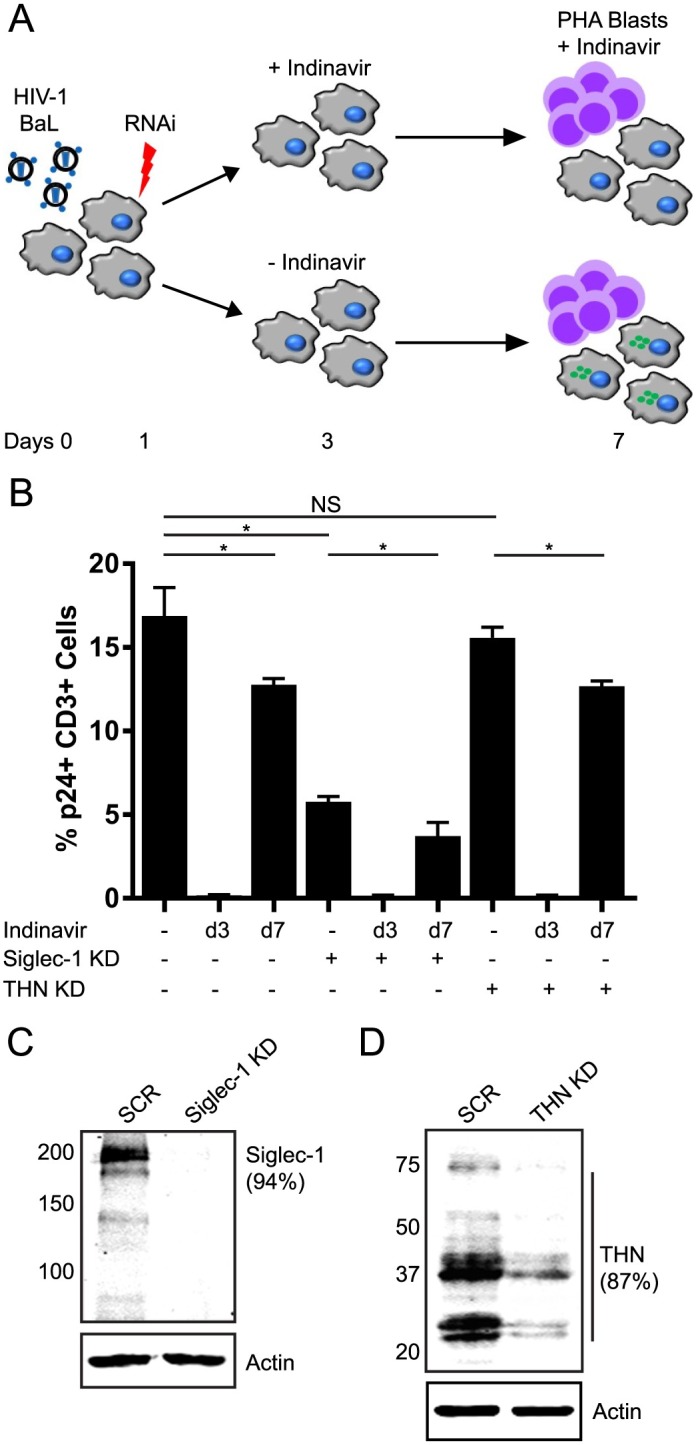
Role of Siglec-1 in macrophage-T cell transmission. **(A)** Schematic outline of transmission experiment. HIV-1 BaL was employed to infect MDMs at an MOI of 3 (day 1). The next day the cells were treated with inhibitory RNAs specific for Siglec-1 or tetherin. On day 3, indinavir was added to one set of cells to inhibit viral production. On day 7 post-infection, indinavir or control media was added 2 hours prior to the addition of autologous CD4+ T cells. The macrophage-T cell coculture was maintained for 12 hours prior to separation of T cells and culture of these cells apart from MDMs for an additional 24 hours in the presence of indinavir. The efficiency of virus transmission was assessed by staining for CD3 and p24 and counting via flow cytometry. **(B)** Results of transmission in each experimental arm, indicating the % of total CD3+ cells staining for p24. Error bars indicate standard deviations from three separate experiments. NS = not significant; * = P <0.05. **(C)** Western blot indicating Siglec-1 knockdown (day 6 post-siRNA addition). **(D)** Western blot indicating tetherin knockdown (day 6 post-siRNA addition).

The significance of Siglec-mediated virion uptake into the VCC was next investigated. Macrophages were infected with HIV-1_BAL_, followed by siRNA-mediated depletion of either Siglec-1 or tetherin on the following day. We added indinavir as a control at early timepoints (day 3) as a means of preventing production and accumulation of infectious virus in the VCC. After 7 days of infection, autologous CD4+ T cells were added (3 cells/MDM), and transmission allowed to proceed for an additional 12 hours. Indinavir was then added 2 hours prior to macrophage-T cell co-culture to cells that had not received indinavir at day 3, in order to prevent transmission occurring through new virion formation during the period of cell-cell contact. T cells were then separated from macrophages and stained for CD3 and p24 and counted by flow cytometry. A schematic of this experiment is presented in Fig 9A. Addition of early (day 3) indinavir prevented transmission events in each group (Fig 9B, d3 lanes). Remarkably, Siglec-1 knockdown significantly reduced transmission (Fig 9B, compare (-) indinavir bar with and without Siglec-1 depletion, and compare d7 indinavir with and without Siglec-1 depletion). Tetherin knockdown in this experiment did not have any significant effect on transmission (due to the potent *vpu* allele of BaL as will be discussed below). In each group, late indinavir addition reduced transmission, indicating that some new virus formation during the contact period contributed to transmission events. However, the majority of the virus transmitted in these experiments came from pre-formed virions within the macrophage. We conclude that Siglec-mediated internalization of virions into the VCC plays a significant role in VCC formation, and that virions retained in the VCC can subsequently contribute to transmission to T cells.

## Discussion

HIV-1- infected macrophages demonstrate prominent intracellular compartments filled with virions (VCCs). These compartments have been postulated to be HIV-1 assembly sites and feature tubular connections with the plasma membrane. However, VCCs also share many features of the viral storage compartment in uninfected monocytoid DCs (mDCs). It has become increasingly clear in recent years that particle capture by mDCs is mediated by the cell surface lectin Siglec-1/CD169 [19, 26]. Siglec-1 interacts with gangliosides on the virion lipid envelope, to mediate particle capture and internalization in mDCs [19, 20, 26]. The major ganglioside involved in HIV-1 particle capture events is GM3, although others such as GM1 may also play a role [19, 21]. Siglec-1-mediated particle capture is also a prominent feature of macrophages, where it similarly facilitates particle capture and trans-infection of T lymphocytes in the case of HIV-1 or B lymphocytes in the case of MLV [23–25]. Here we confirm that Siglec-1-mediated particle capture leads to internalization of exogenous virus-like particles or infectious virions into human macrophages, and show that the internalized particles and Siglec-1 colocalize with known markers of the VCC. Depletion of Siglec-1 led to markedly diminished formation of VCCs within infected macrophages, suggesting that the majority of virions within the VCC of infected macrophages are formed peripherally and then are internalized together with Siglec-1 to this compartment.

Siglec-1 moved together with VLPs toward the perinuclear region of macrophages, sometimes accompanied by the formation of narrow Siglec-1 and VLP+ tubules, eventually becoming highly concentrated deep in the cell. The compartment to which VLPs were delivered was proven to be identical to the VCC, as added VLPs colocalized with mature virions in the infected cells and with typical VCC markers. This argues for a common internalization pathway that brings particles formed in an infected macrophage into the VCC and is accessible to exogenous particles. We suggest that this common pathway is formed through a macropinocytosis-like process, and that the common element involved in determining the location of both the endogenous particles from the infected cell and the exogenous VLPs is Siglec-1. Macropinocytosis of HIV-1 virions into macrophages has been previously described [30, 31]. This same process is likely to occur when particles bud from infected macrophages, as Siglec-1 is found highly concentrated in VCCs without adding any VLPs exogenously. Our data support a model in which Siglec-1 attaches to gangliosides, most prominently GM3 [19], on the virion envelope during the budding process on the plasma membrane, followed by internalization of the virion-Siglec-1 complex along a tubular assembly and into the VCC. Thus we propose that the connections observed previously leading from the VCC to the plasma membrane [5, 8, 9] are likely the conduits of virion internalization, rather than exit, for HIV-1.

The site of assembly in macrophages has been debated. Immature and budding particles can sometimes be seen within VCCs in electron micrographs, and this provides visual evidence that budding can occur into the VCC [6, 7, 10]. Our data do not contradict these observations. However, the finding that exogenous VLPs concentrate Siglec-1 and move rapidly from the plasma membrane to the VCC suggests that the plasma membrane is likely to be the major site of assembly in macrophages. Because the internalization of virions occurs within minutes, any static imaging analysis of infected macrophages in culture will visualize a predominance of virion particles in the VCC, rather than on the plasma membrane. Quantification of the amount of assembly and budding from the macrophage plasma membrane versus potential assembly on intracellular membranes of the VCC will require future dynamic imaging studies in which internalization of captured virions is included in the analysis.

The significance of Siglec-1-mediated capture of virions by macrophages and of VCC formation itself is most likely in providing a storage or transport compartment for infectious virions that mediate trans-infection of CD4+ T lymphocytes. Data presented here indicate that formation of the VCC can be mediated by infection of the macrophage, or alternatively by uptake of exogenous virions into an identical compartment in uninfected macrophages. The fact that either route can lead to VCC formation and mediate infection of T cells raises interesting questions relevant to HIV transmission and pathogenesis in humans. The majority of transmitted HIV-1 isolates are not truly macrophage-tropic, as defined by the ability to infect macrophage-like cells bearing low amounts of surface CD4. However, such isolates arise later in infection in a number of tissues such as the central nervous system [32]. We postulate that early in infection, Siglec-1-mediated capture of virions that are inefficient in infecting macrophages can lead to VCC formation and contribute to trans-infection of T cells, while macrophage populations that become infected as macrophage-tropic viruses evolve within an individual form VCCs bearing their “own” viruses. In both scenarios, the captured virions may be at least transiently protected from immune surveillance and from neutralization [13, 14]. Our results also raise the interesting possibility that some VCCs may bear a mixture of particles, some arising from the infected macrophage and some captured from surrounding cells and tissues, having originated from other infected cells.

**Fig 10:**
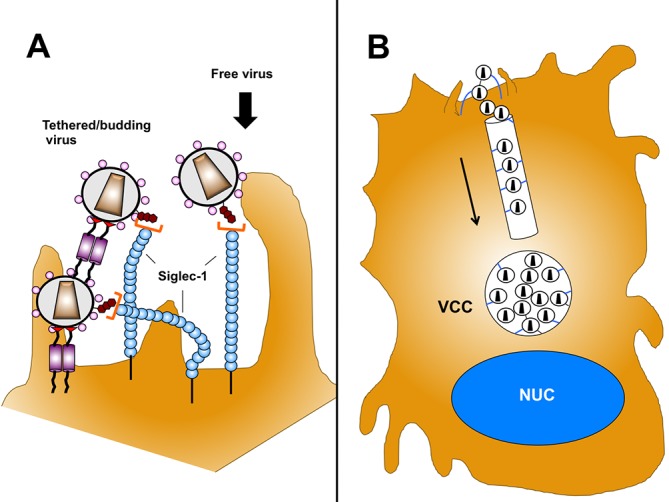
Model for roles of Siglec-1 and tetherin in capture and internalization of HIV-1 virions to the VCC. **(A)** Siglec-1 proteins are shown binding to GM3 gangliosides on virion envelope. Tetherin is indicated in the chain on the left, not to scale, illustrating how retention by tetherin followed by capture/internalization by Siglec-1 may occur. Free virions arising from the infected cell or from the surrounding environment are also captured by Siglec-1 (right). **(B)** Following capture by Siglec-1, virions are internalized and co-transported with Siglec to the VCC. Shown is a single channel leading to the VCC.

What is the role of tetherin in this process? Tetherin has been noted to inhibit transmission from myeloid cells to T cells in some studies [15, 16, 33] while having more variable effects in others [34]. We propose a model in which tetherin restricts release of virus at the plasma membrane, and then Siglec-1 interaction with GM3 on the virion membrane *in cis* leads to internalization of the retained virions (Fig 10). Tetherin and Siglec-1 subsequently both are internalized together with the retained virions to the VCC, where they are readily seen to colocalize. This model provides an explanation for the finding that knockdown of either Siglec-1 or tetherin leads to diminished volumes of the VCC. We found that Siglec-1 knockdown was somewhat more potent in reducing VCC volume than tetherin knockdown, suggesting a very prominent role in this process. The relative role of tetherin may be affected by the activation state of the macrophage, as well as by the activity of the particular Vpu protein expressed by the infecting virus. Neil and colleagues have shown that there is a viral allele-specific variation in the ability of Vpu to downregulate tetherin [27], and we found that Vpu from NL4.3 was only partially effective at tetherin downregulation in MDMs [15]. Thus when a *vpu* allele is potent, the role of tetherin in VCC formation and its negative effect in transmission can be largely negated. This explains differences observed in viral transmission following tetherin depletion in the present study (no apparent effect upon transmission of virus expressing a potent *vpu* allele) in contrast with our prior findings using NL4.3 or NLUdel (enhanced transmission of infection upon tetherin depletion)[15]. Siglec-1-mediated virion capture during particle budding, on the other hand, is not altered by Vpu, and therefore Siglec-1-mediate effects on VCC formation and transmission of virus is preserved regardless of the presence or absence of a potent *vpu* allele. Siglec-1 may be a more important contributor to VCC formation than tetherin in the setting of primary HIV-1 isolates, as most of these isolates will encode a *vpu* allele that is more active than that of NL4.3 [27]. Another difference is that Siglec-1 is able to mediate capture of exogenous virions or of endogenous virions and subsequently generate VCCs, whereas tetherin can only contribute to the retention and capture of virions arising from the infected cell membrane.

We note that although both tetherin and Siglec-1 contribute to VCC formation, they play opposing roles in cell-cell transmission. Tetherin plays a negative role in macrophage-to-T cell transmission when it is not downregulated by Vpu or depleted by siRNA [15, 16]. Siglec-1, on the other hand, captures virions and leads to their internalization into the VCC in a manner that allows and perhaps facilitates subsequent transmission events. It will be interesting to dissect more completely how Siglec-1-mediated capture allows transmission to occur at the virologic synapse, while tetherin does not.

If the VCC serves as a reservoir in long-lived tissue-resident macrophages, then strategies designed to eradicate HIV will need to target this compartment. Because the Siglec-1-GM3 interaction is common to both the intracellular compartment in DCs and in macrophages, a common strategy could potentially target HIV captured by both cell types. Delivery of an inhibitory agent to the VCC of macrophages or to the DC could serve the dual function of eradicating a potential reservoir and preventing trans-infection of T cells. Pursuit of strategies targeting this common pathway in HIV-infected individuals are warranted.

## Materials and methods

### Ethics statement

Human blood for the preparation of monocyte-derived macrophages and other experiments in this work was obtained from volunteer donors and was de-identified prior to handling by the investigators. Informed consent was obtained from participants. Blood was collected under a protocol approved by the Emory Institutional Review Board.

### Isolation and maturation of monocyte-derived macrophages

Human peripheral blood mononuclear cells (PBMCs) were isolated from fresh heparinized blood by Ficoll-Hypaque gradient centrifugation. PBMCs from buffy coats were pooled and extensively washed to remove platelets. Monocytes were enriched by magnetic-labeling using Monocyte Isolation Kit II (Miltenyi Biotec Inc) according to manufacturer’s protocol. Enriched monocytes were adhered to poly-D-lysine coated plates (Corning) and 35 mm MatTek dishes (MatTek Corporation). Monocytes were maintained in RPMI-1640 supplemented with 10% FBS, 100 ug/ml streptomycin, 100 U/ml penicillin, 2 mM glutamine and 5 ng/ml GM-CSF or 20 ng/ml M-CSF (R&D Systems). Monocytes cultures were maintained in cytokine supplemented media for 7 days to facilitate maturation into monocyte-derived macrophages (MDMs). Media was replaced every 2–3 days. Macrophage purity was assessed by CD14 staining on day 8.

### p24 ELISA

P24 content of HIV-1 Gag-EGFP virus-like particles (VLPs) from stocks and MDM cell lysates were measured using a p24 antigen capture ELISA. Accurate p24 measurement of immature HIV-1 VLPs requires raising SDS level of lysis solution to 0.5% and heating for 10 min at 60°C. Murine anti-p24 capture antibody 183-H12-5C (CA183) was obtained from Bruce Chesebro and Kathy Wehrly through the NIH AIDS Research and Reference Reagent Program. CA183 was coated onto 96-well plates at a dilution of 1:2000 in PBS and incubated overnight at 37°C. Plates were blocked for 1 hour at 37°C with 5% fetal calf serum in PBS. The detection of bound p24 was determined using HIV-Ig, obtained from NABI through the NIH AIDS Research and Reference Reagent Program, at a dilution of 1:20,000 for 1 hour at 37°C. Colorimetric analysis was performed using the Immunopure TMB Substrate Kit (Pierce, Rockford, IL) and absorbance was read at 450 nm. Recombinant p24 was used for the standard curve and sensitive to less than 20 pg of p24.

### Siglec-1 and tetherin RNAi

Siglec-1 (HSS110029), tetherin (HSS101115) and negative control Med GC Stealth siRNAs were obtained from Life Technologies (Grand Island, NY). For knockdown experiments, MDMs were transfected with 60 nM Stealth siRNAs using Lipofectamine RNAiMax (Life Technologies) according to manufacturer’s protocols. On the following day, transfection complex containing media was removed and cells washed once with complete media. Samples were collected at various time points post-transfection and either stored at -80°C until analysis or further processed for immunofluorescence microscopy as described.

### Flow cytometry

Both M-CSF and GM-CSF matured MDMs were assayed for Siglec-1 and tetherin cell surface concentrations in the presence or absence of 1000 U/ml Universal Type I IFN Alpha (PBL Assay Science). Mature MDMs were stimulated overnight with IFN, cells washed with PBS and detached using Versene (Life Technologies) with gentle scraping. Surface CD14 (BD Pharmingen, Cat. No. 555399), sheep anti-Siglec-1 (R&D Systems, Cat. No. AF5197) and tetherin staining procedures were performed as previously described [35]. FACS Canto II flow cytometer (BD Biosciences) and FlowJo software (Treestar Inc) were used for analyses.

### Viral stock generation and MDM infection

pNL4-3 proviral plasmid was obtained through the NIH AIDS Reagent Program, Division of AIDS, NIAID, NIH; from Malcolm Martin. pNLUdel proviral plasmid [36] was obtained from Klaus Strebel, NIAID, NIH. pHCMV-G [37] for VSV-G expression was obtained from Jane Burns at UC San Diego. Vesicular stomatitis virus g glycoprotein (VSV-G)-pseudotyped or wild type HIV-1 NL4.3 and NLUdel stocks were created by transfection of 293T cells (CRL 3216 from American Type Culture Collection, ATCC) using jetPRIME (Polyplus) transfection reagent according to manufacturer’s instructions. Virus was harvested from transfected cell supernatants at 36 hours post-transfection, clarified, filtered through a 0.45-μm filter and stored at -80°C. Primary HIV-1 isolate BaL stocks were prepared as follows: Human peripheral blood mononuclear cells (PBMCs) were isolated from fresh heparinized blood by standard Ficoll-Hypaque gradient centrifugation methods. PBMCs were resuspended in RPMI 1640 supplemented with 20% heat-inactivated fetal bovine serum and 50 μg/ml gentamicin (RPMI 1640-GM). Primary HIV-1 isolates were propagated in PBMCs stimulated with 5 μg/ml phytohemagglutinin (PHA) and 5% interleukin 2 (IL-2). The IL-2/PHS-stimulated cells were infected using a high-titer seed stock of virus minimally passaged in PBMCs, starting from a viral stock obtained through the NIH AIDS Reagent Program (from Dr. Suzanne Gartner, Dr. Mikulas Popovic and Dr. Robert Gallo). One ml of virus was transferred to the flask containing freshly stimulated PBMCs and incubated overnight at 37°C in 5% CO2. The cells were washed extensively and resuspended in 30 ml of RPMI-GM with IL-2. Typically, the virus was harvested two times; the first harvest was on day 4 post-infection, with subsequent harvest on day 7. The virus-containing supernatants were collected, clarified by centrifugation, and filtered through a 0.45-μm filter. The virus was then aliquoted into 1-ml sterile screw-cap cryovials and stored at -80°C. Infectivity of viral stocks were assayed for infectivity using TZM-bl indicator cells (obtained through the NIH AIDS Reagent Program, Division of AIDS, NIAID, NIH; from Dr. John C. Kappes, Dr. Xiaoyun Wu and Tranzyme Inc.). TZM-bl were incubated for 48 hours, and 100 μl of supernatant was removed from each well prior to the addition of 100 μl of Bright Glo substrate (Promega, Madison, WI). Measurement of infectivity involved transfer of 150 μl of cell/substrate mixture to black 96-well solid plates and measurement of luminescence using a Packard TopCount luminometer. MDMs were infected with VSV-G-pseudotyped HIV-1 at a TCID50 of 1–2/cell and primary HIV-1 isolate BaL at 0.5 TCID50/cell. The exception to this is the experiment in which a higher MOI of input virus was utilized to assess for uptake of infectious virions by Siglec-1. In this experiment, MDMs were infected/exposed to 100ng/1 x 10^5^ cells of NLΔEnv virus pseudotyped with BaL Env in the presence or absence of 10 μM BMS-626529 (Aurum Pharmatech, Catalogue number W-5929). NLΔEnv/BaL stocks were prepared by transfection of 293T cells as outlined above using pNLEnv-1 proviral plasmid [38] from Klaus Strebel and BAL.26 Env pseudotyping construct [39] from David Montefiori, Duke University.

### Immunofluorescence microscopy

5.0 x 10^5^ MDMs were seeded on Collagen-I coated 35 mm MatTek dishes (MatTek) and allowed to mature for a minimum of 7 days as described. At the appropriate time, MDMs were fixed with 4% paraformaldehyde (PFA) in sodium phosphate buffer (PBS) for 10 min, permeabilized with 0.2% Triton X-100, and blocked with Dako blocking buffer (Dako) supplemented with 6 μg/ml human IgG. Cells were incubated with combinations of rabbit anti-tetherin antisera [35], murine anti-siglec-1 (AbD Serotec, clone 7–239), murine anti-p24 (Beckman Coulter, KC57-FITC or RD1), or murine anti-CD9 (BD Pharmingen, Cat. No. 555370) in DAKO Background-Reducing Antibody Diluent, washed thoroughly, and incubated with the appropriate secondary antibodies. Immunostaining requiring a murine primary and the directly conjugated murine anti-p24 KC57 FITC were performed as follows. Primary murine anti-CD9 or anti-Siglec-1 labeling was performed as previously described. MDMs were then blocked with 6 μg/ml murine IgG in DAKO Background-Reducing Antibody Diluent for 1 hour, washed and then immunostained with anti-p24 KC57-FITC in DAKO Background-Reducing Antibody Diluent supplemented with 6 μg/ml murine IgG. In order to visualize the nucleus, cells were subsequently stained with DAPI (4′,6′-diamidino-2-phenylindole) at 300 nM in PBS for 15 minutes at room temperature, washed several times with PBS, and imaged. Immunofluorescence images were acquired using a DeltaVision RT deconvolution microscope (Applied Precision/GE Life Sciences), and data analyses were performed with Volocity 6.3 software (Perkin-Elmer). Immunofluorescence colocalization was calculated using stringent image thresholding and confirmed by visual object identification methods [40]. For most colocalization analyses reported here, five representative images were quantified. For the volume measurements of the VCC, 30 images were quantified for each experimental arm.

### Time-lapse imaging of virus containing compartment formation in MDMs

3D time-lapse live cell imaging was carried out with a Zeiss LSM780 confocal microscope using a C-Apo 40x/1.2NA water-immersion objective. A suitable field of view was selected, and full cell volume was imaged by acquiring 8–12 Z-stacks spaced by 1 μm every 2.5 minutes, using a minimal power of 405 and 488 nm lasers for Hoechst-33342, Gag-EGFP VLPs respectively. The DefiniteFocus module (Carl Zeiss) was utilized to correct for axial drift. Imaging was done at 37°C using the Zeiss environmental chamber maintained at 5% CO_2_. A single Z-plane of the cells showing VCC formation was converted into each of the movies shown.

### Transmission electron microscopy

MDMs were cultured on poly-D-lysine (PDL) coated ACLAR embedded film (Ted Pella; Redding, CA) as described. MDMs were harvested 10 days post-infection unless indicated otherwise, fixed in 2.5% PFA and 2.5% glutaraldehyde for 2 hours followed by embedding in Epon. Serial 100 nm sections were stained with heavy metals and images were obtained using a Hitachi H-7500 transmission electron microscope at 120 kV.

### VLP production and MDM capture assay

HIV-1 Gag-EGFP VLPs were generated by transient transfection of HEK 293T cells with pVRC-3900 and pVRC/GAGOPT-GFP at a ratio of 3:1 respectively. For experiments where morphologically aberrant VLPs were desired a ratio of 1:1 was used. The HIV-1 Pr55Gag construct, pVRC-3900, is an expression plasmid encoding a codon-optimized HIV-1 Pr55Gag polyprotein and was kindly provided by Gary Nabel (VRC, NIH)[41]. A c-terminal EGFP fusion Pr55Gag construct, pVRC/GAGOPT-GFP, was generated by PCR amplification of the Pr55Gag region from pVRC-3900 and subsequent subcloning into the HindIII/BamHI sites of pEGFP-N3. VLPs were harvested 48 hours post-transfection, supernatants clarified, filtered through a 0.45 μm filter and concentrated through a 20% sucrose cushion. VLP pellets were resuspended in ice cold PBS and stored at -80°C. Additionally, HIV-1 Gag-EGFP VLP stocks were produced in HEK 293T cells pre-treated for 2 days and throughout VLP production with 10 μM PDMP (Calbiochem). PDMP (1-phenyl-2-decanoylamino-3-morpholino-1-propanol) inhibits the activity of glucosylceramide synthase (UDP-glucose:ceramide glucosyltransferase) which initiates the biosynthesis of animal gangliosides (GSLs).

HIV-1 Gag-EGFP VLP capture and internalization assays were performed on GM-CSF matured MDMs from between day 7 to 10 post-plating in 35 mm Collagen-I coated MatTek dishes. MDMs were incubated with 100 ng of HIV-1 Gag-EGFP VLPs/1.0x10^5^ cells in plain RPMI media in a 37°C/5% CO_2_ for times indicated. MDMs were washed extensively with PBS prior to either cell lysis for p24 quantification by ELISA or fixation and immunofluorescent staining.

Sialic acid removal from HIV-1 Gag-EGFP VLP membrane associated glycosphingolipids was performed by incubating sucrose-purified VLPs with 1.0 U/μl of a neuraminidase (NA) (NEB; P0728S). This enzyme is a specific exoglycosidase that hydrolyzes α2–3 N-acetyl-neuraminic acid residues, and exhibits a 260-fold preference for α2–3 sialyl linkages versus α2–6 sialyl linkages while exhibiting only trace hydrolysis of α2–8 sialyl linkages. VLPs were treated with neuraminidase in PBS for 6 hours at 37°C. In order to assess efficiency of sialic acid removal, 100 ng of both mock and NA-treated VLPs were incubated on poly-D-lysine coated 35 mm MatTek dishes for 1 hour at room temperature. The solution was then aspirated and washed several times prior to fixation with 4% paraformaldehyde. VLPs were then stained with wheat germ agglutinin conjugated with Alexa Fluor 647 (Life Technologies, W32466). VLPs from analyzed preparations were also used for MDM uptake experiments.

### Macrophage-T cell trans-infection assay

MDMs were GM-CSF matured for seven days on Poly-D-Lysine coated 12-well plates (Corning) prior to overnight infection at 3 TCID50/cell with a biological stock of HIV-1 BaL. Following day knockdown of Siglec-1 and tetherin were performed as previously described. Co-cultures in some instances were treated with 1 μM Indinavir Sulfate (reagent obtained through the NIH AIDS Reagent Program, Division of AIDS, NIAID, NIH) at either 3 days post-infection or 2 hours prior to CD4^+^ T cell addition on day 7. On day 7 post-infection, autologous CD4^+^ T cells were added at 3 cells/MDM in complete medium. Co-cultures were incubated for 12 h at 37°C and 5% CO_2_. CD4^+^ T cells were isolated from MDMs using Versene solution (Thermo Fisher Scientific), washed in serum-free RPMI-1640 and cultured for 24 h in complete medium supplemented with 5 ng/ml rhIL-2 (R&D Systems) and 1 μM indinavir sulfate. CD4^+^ T cells were then fixed and permeabilized using Fixation/Permeabilization Solution Kit (BD Biosciences, San Jose, CA) prior to staining with anti-human CD3-APC (Cat. No. 555342, BD Biosciences) and anti-HIV-1 p24 KC57-FITC (Cat. No. 6604665; Beckman Coulter). Cells were analyzed using a FACSCanto II flow cytometer (BD Biosciences).

### Statistical analyses

All graphical data are presented as means +/- SD. Statistical significance between groups was determined by unpaired *t* test using GraphPad Prism 4.02. Significant P values <0.05 are noted within figures.

## Supporting information

S1 Fig Quantitation of depletion of GM3 on VLPs following neuraminidase treatment. **(A)** Representative images are shown of sucrose purified HIV-1 Gag-EGFP VLPs treated with or without neuraminidase. VLPs were added to PDL coated MatTek dishes at RT in PBS for 1 hr, followed by washing and 4% PFA fixation. Samples were then labeled with Alexa Fluor 647 conjugated-wheat germ agglutinin for 30 min. Size bar = 5 μm. **(B)** Quantification of HIV-1 Gag-EGFP VLPs and associated 647 signal intensity using the Volocity 6.3 measurement module. Data for more than 15,000 HIV-1 Gag-EGFP VLPs plotted as percent MFI 647/GFP. GFP positive areas exceeding 500 nm were excluded from analysis.
(TIF)

S2 Fig Tetherin depletion in MDMs. MDMs were transfected with 60 nM control or tetherin siRNA on day 8 after plating. Cell lysates were harvested and analyzed by Western Blotting for tetherin and actin expression at indicated time points. Control (scrambled, SCR) tetherin blot from day 1 is shown on the left.
(TIF)

S3 Fig Siglec-1 depletion reduces VCC in VLP-exposed MDMs. **(A)** MDMs exposed to control siRNA were examined for EGFP fluorescence representing VLPs (green), Siglec-1 (red), and DIC. **(B)** MDMs following siRNA-mediated depletion of Siglec-1 were examined for collections of VLPs (green), Siglec-1 (red), and by DIC. **(C, D, E)** P24 volume/cell from 15 MDMs exposed to EGFP VLPs from each group were quantified using the Volocity 6.3 measurement module. Mean +/- SD shown for each RNAi group. **(C)** 30 minutes. **(D)** 2 hours. **(E)** 6 hours.
(EPS)

S4 Fig Siglec-1 RNAi reduces VCC formation in HIV-1 BaL-infected MDMs. MDMs were transfected with 60 nM scrambled, Siglec-1 or tetherin siRNA followed by subsequent next day infection of primary HIV-1 isolate BaL at TCID50 0.5/cell. At day 10 post-infection, MDMs were washed, fixed with 4% PFA and immunostained for p24 (green), Siglec-1 (red), tetherin (magenta) and DAPI co-stained. Size bars = 10 μm.
(TIF)

S5 Fig Anti-p24 mAb KC57-RD1 fails to recognize immature HIV-1 Gag-EGFP VLPs internalized by MDMs. 400 ng of HIV-1 Gag-EGFP were added to MDM cultures in MatTek dishes and allowed to internalize for 2 hours. MDMs were then washed, fixed in 4% PFA, immunostained with anti-p24 (red, KC57-RD1) and DAPI co-stained. Two representative fluorescent micrographs shown. Size bars = 10 μm.
(TIF)

S6 Fig BMS-626529 prevents HIV-1 infection and does not diminish p24 and Siglec-1 colocalization in virus-exposed MDMs. **(A)** Dilutions of NL delta Env virus pseudotyped with BaL Env (NLΔE/BaL) were pre-treated with either DMSO or 10 μM BMS-626529 and infectivity assessed on TZM-bl cells. **(B)** Percentage of p24/Siglec-1 colocalization in 17 thresholded images of NL NLΔE/BaL-infected MDMs from DMSO and BMS-626529 groups. **(C)** Percentage of Siglec-1/p24 colocalization in 17 thresholded images of NL NLΔE/BaL infected MDMs from DMSO and BMS-626529 groups. **(D)** P24 VCC volume/cell for 17 NLΔE/BaL infected MDMs from DMSO and BMS-626529 groups. Quantitation created using Volocity 6.3 measurement module. Mean +/- SD shown for each group.
(EPS)

S7 Fig Electron microscopic imaging of infectious viral particle uptake into VCCs. BaL-pseudotyped NLΔEnv was added at a TCID50 of 120 (as in Fig 8) to MDM cultures grown on ACLAR coverslips in the presence or absence of 10 μM BMS-626529. After 24 hours, samples were fixed, embedded, and sectioned for transmission EM. (A) MDMs in absence of BMS-626529. Section indicating VCC with dashed box. (B) Higher power view of dashed area from (A). (C) VCCs from MDM exposed to virus in presence of BMS-626529. (D) Higher power view of dashed area from (C). Size bars = 0.5 μM.
(EPS)

S1 Movie Time-lapse imaging of capture and uptake of HIV-EGFP VLPs into the VCC of a single MDM. (MP4)

S2 Movie Time-lapse imaging of capture and uptake of HIV-EGFP VLPs into VCCs of two MDMs. (MP4)
