# Supplementary figures and images for: Siglec-1 initiates formation of the virus-containing compartment and enhances macrophage-to-T cell transmission of HIV-1
Source: PLoS Pathog. 2017 Jan 27;13(1):e1006181. doi: 10.1371/journal.ppat.1006181 (PMC5298340; doi:10.1371/journal.ppat.1006181)

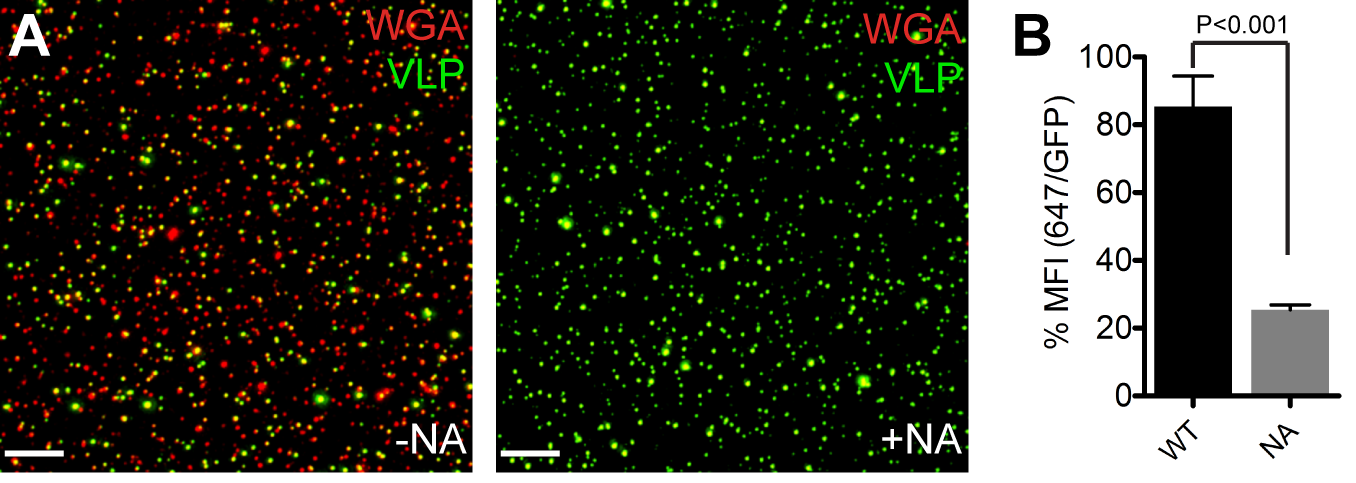

Supplement: S1 Fig — (A) Representative images are shown of sucrose purified HIV-1 Gag-EGFP VLPs treated with or without neuraminidase. VLPs were added to PDL coated MatTek dishes at RT in PBS for 1 hr, followed by washing and 4% PFA fixation. Samples were then labeled with Alexa Fluor 647 conjugated-wheat germ agglutinin for 30 min. Size bar = 5 μm. (B) Quantification of HIV-1 Gag-EGFP VLPs and associated 647 signal intensity using the Volocity 6.3 measurement module. Data for more than 15,000 HIV-1 Gag-EGFP VLPs plotted as percent MFI 647/GFP. GFP positive areas exceeding 500 nm were excluded from analysis. (TIF) [file ppat.1006181.s001.tif]

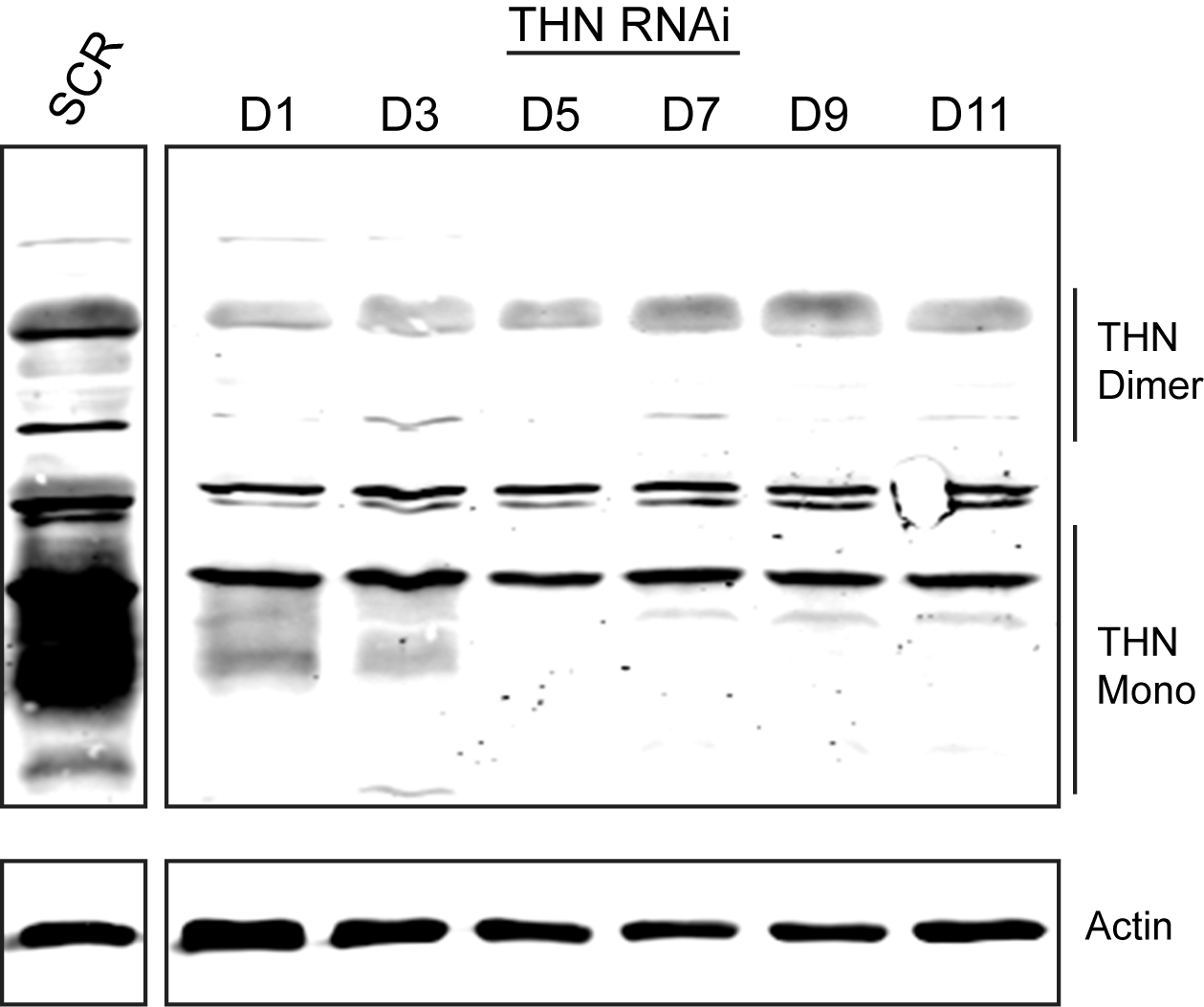

Supplement: S2 Fig — MDMs were transfected with 60 nM control or tetherin siRNA on day 8 after plating. Cell lysates were harvested and analyzed by Western Blotting for tetherin and actin expression at indicated time points. Control (scrambled, SCR) tetherin blot from day 1 is shown on the left. (TIF) [file ppat.1006181.s002.tif]

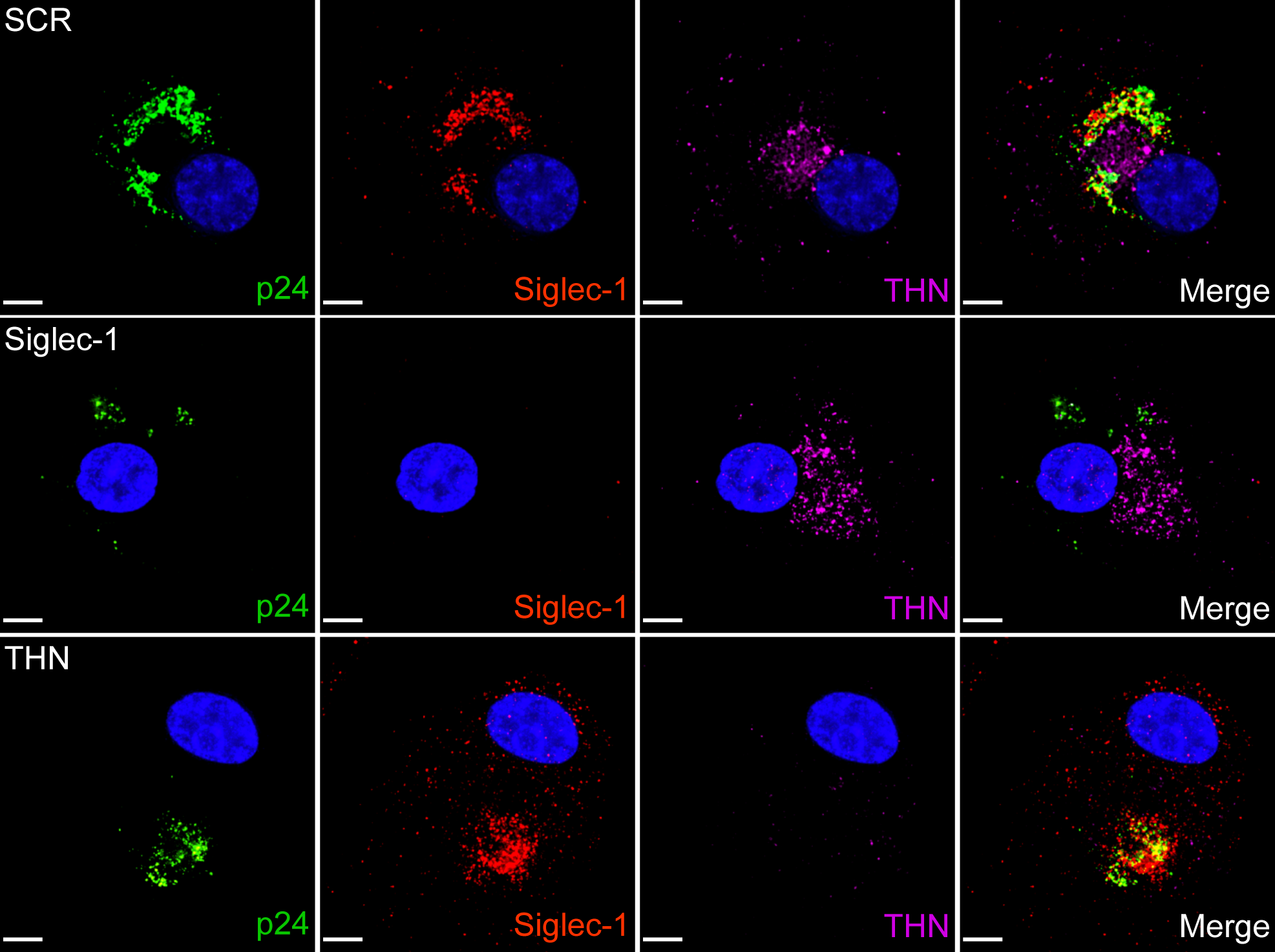

Supplement: S4 Fig — MDMs were transfected with 60 nM scrambled, Siglec-1 or tetherin siRNA followed by subsequent next day infection of primary HIV-1 isolate BaL at TCID50 0.5/cell. At day 10 post-infection, MDMs were washed, fixed with 4% PFA and immunostained for p24 (green), Siglec-1 (red), tetherin (magenta) and DAPI co-stained. Size bars = 10 μm. (TIF) [file ppat.1006181.s004.tif]

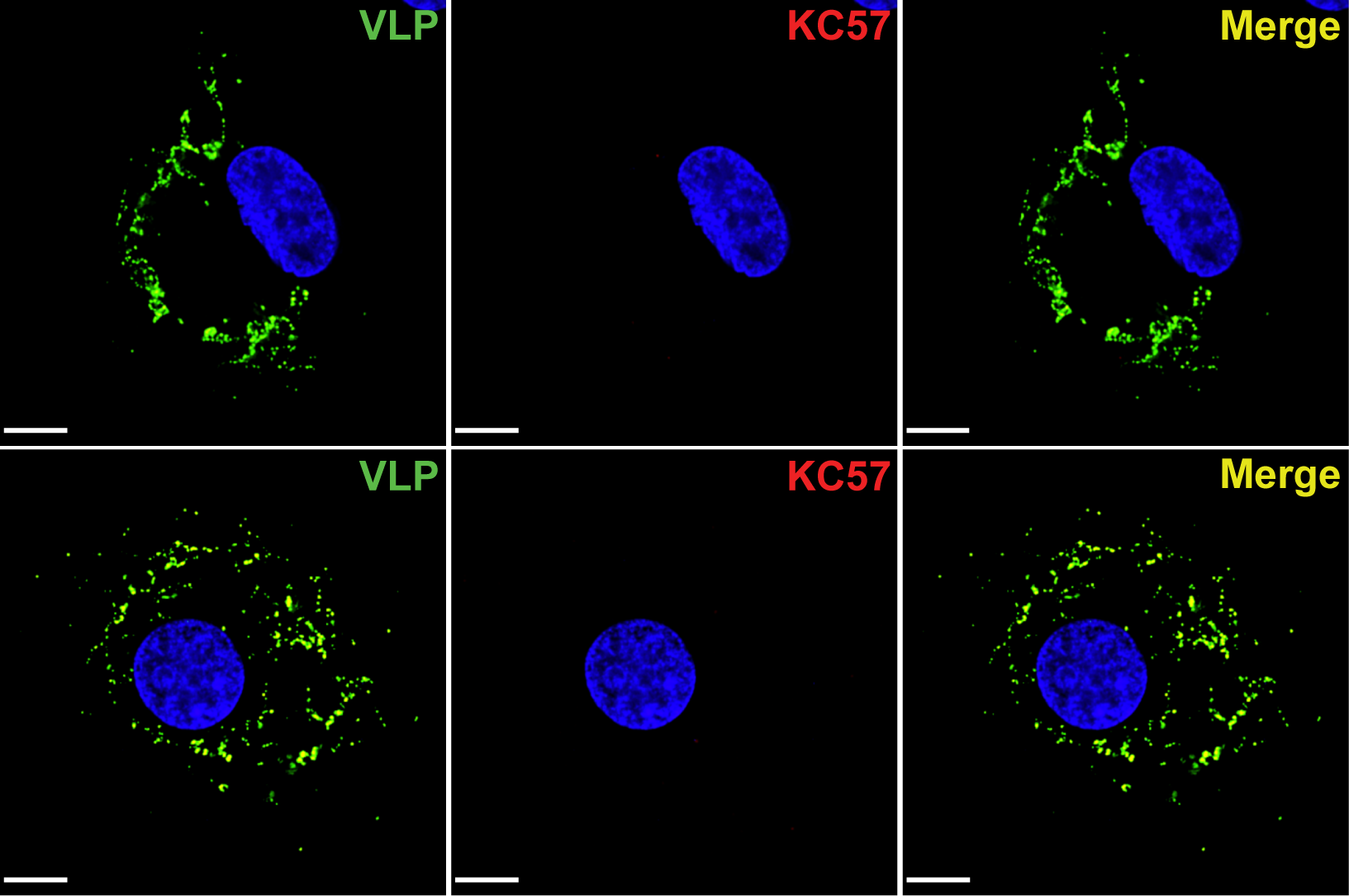

Supplement: S5 Fig — 400 ng of HIV-1 Gag-EGFP were added to MDM cultures in MatTek dishes and allowed to internalize for 2 hours. MDMs were then washed, fixed in 4% PFA, immunostained with anti-p24 (red, KC57-RD1) and DAPI co-stained. Two representative fluorescent micrographs shown. Size bars = 10 μm. (TIF) [file ppat.1006181.s005.tif]
